# Biophotonics in oral health: bridging light-based innovation and clinical practice

**DOI:** 10.3389/froh.2026.1978161

**Published:** 2026-10-28

**Authors:** R. J. Bensadoun, K. Lairedj, B. R. Carlotti

**Affiliations:** RJB-Consulting, Nice, France

**Keywords:** biophotonics, dosimetry, oral health, oral mucositis, photobiomodulation, precision medicine

## Abstract

Biophotonics is reshaping oral healthcare by transitioning light-based technologies from isolated procedural applications toward integrated diagnostic, therapeutic, and regenerative strategies. Among these approaches, photobiomodulation (PBM) therapy has achieved substantial clinical visibility due to its capacity to modulate inflammation, pain, mitochondrial metabolism, vascular responses, and tissue repair without producing destructive thermal effects. While oral and oropharyngeal mucositis represent the most clinically and quantitatively mature models for PBM, its applications now encompass salivary dysfunction, periodontal therapy, oral surgery, temporomandibular disorders (TMD), implant osseointegration, orthodontics, and inflammatory mucosal conditions. However, translating positive biological outcomes into routine practice remains limited by substantial heterogeneity in device architectures, wavelengths, beam profiles, and reporting standards. This comprehensive narrative review examines the physical, clinical, and dosimetric landscape of oral biophotonics. We address critical gaps in current literature—including reporting shortfalls, negative clinical trials, and general safety risks—and outline the technological evolution toward target-tissue dosimetry. By integrating computational photon-transport models, biological monitoring, and artificial intelligence-assisted dosimetry, we propose a transitional framework toward precision oral photomedicine, wherein anatomical and optical tissue properties guide individualized, patient-centered light delivery.

## 1 Introduction

Light has long occupied a distinctive place in dentistry and oral medicine, serving applications in visualization, polymerization, surgery, ablation, disinfection, fluorescence-based detection, photodynamic approaches, and, increasingly, biological modulation (1–3). Photobiomodulation (PBM) therapy, historically termed low-level laser (or light) therapy (LLLT), represents one of the most clinically developed therapeutic branches of this field (1, 3). PBM utilizes non-ionizing radiation in the visible red and near-infrared (NIR) spectrum to induce nonthermal biological responses through mechanisms involving mitochondrial metabolism, redox signaling, nitric oxide production, intracellular calcium modulation, transcriptional regulation, and tissue repair (1, 4–8).

Oral tissues provide an exceptionally favorable environment for therapeutic biophotonics due to their accessibility, high vascularization, and rapid biological turnover. Concurrently, the oral cavity presents substantial optical complexity: mucosal thickness and keratinization vary across sites; saliva, blood, edema, pigmentation, bone, teeth, and restorative materials modify photon propagation; and anatomical curvatures limit homogeneous illumination (9). These features render the oral cavity both an ideal clinical target and an exceptional laboratory for studying precision photomedicine.

### 1.1 Review nature and literature identification

To contextualize the evidence discussed herein, this manuscript is explicitly structured as a comprehensive narrative and perspective review rather than a systematic review. The literature surveyed was identified via targeted searches of the PubMed/MEDLINE, Embase, and Cochrane Library databases for articles published up to mid-2026. Search terms focused on combinations of “photobiomodulation”, “low-level laser therapy”, “biophotonics”, “dosimetry”, and specific oral conditions. Studies were selected illustratively to demonstrate the continuum of evidence, highlight landmark international guidelines, and critically expose the profound methodological and dosimetric heterogeneity that currently limits cross-study comparisons.

The central translational challenge can be summarized as follows: biological efficacy cannot be separated from optical delivery (3). A wavelength, power setting, or nominal fluence specified in a protocol does not accurately define the biological exposure received by the target tissue. Device architecture, beam divergence, source-to-tissue distance, tissue geometry, and optical attenuation are equally critical determinants (3, 9, 10). The purpose of this review is twofold: first, to provide a critically balanced, clinically oriented overview of therapeutic biophotonics across oral health; and second, to examine device design and dosimetry as the core determinants of the next generation of precision oral photomedicine, establishing a valuable conceptual framework for future research and clinical implementation.

## 2 Biological foundations: from photons to oral tissue response

PBM differs fundamentally from ablative or photothermal laser applications; its objective is not tissue removal or coagulation but the modulation of biological processes within a therapeutic optical window. Cytochrome-c oxidase has historically been considered the primary photoacceptor for red and NIR radiation, linking photon absorption to mitochondrial respiration and cellular bioenergetics (6, 7). Contemporary models are broader, encompassing the transient modulation of reactive oxygen species (ROS), nitric oxide (NO) availability, membrane channels, calcium signaling, transcription factors, and immunometabolic pathways (4, 5, 8).

These mechanisms are highly relevant to oral pathologies, which frequently share common biological processes involving:

- Acute and chronic inflammation
- Oxidative stress
- Nociception and impaired microcirculation
- Altered mitochondrial metabolism
- Extracellular matrix remodeling and defective wound healing (2, 11).

PBM can modulate several components of these pathological cascades simultaneously (2, 11). However, this biological breadth requires caution: mechanistic plausibility cannot substitute for indication-specific clinical evidence.

## 3 Oral health applications: a continuum of evidence

### 3.1 Oral mucositis: the benchmark indication and quantitative evidence

Cancer therapy-induced oral mucositis represents the most robust translational model for PBM in oral medicine. Its clinical burden includes severe pain, ulceration, impaired nutrition, dysphagia, opioid dependence, secondary infections, prolonged hospitalizations, and potential interruptions to anticancer regimens (12, 13). Unlike many other oral applications, the clinical positioning of PBM here is anchored by robust quantitative findings and rigorous meta-analyses. Landmark systematic reviews have demonstrated that prophylactic PBM achieves a significant reduction in the risk of developing severe (Grade 3–4) oral mucositis (14–18). For instance, the 2026 systematic review by Pedroso et al (19), evaluating 30 randomized controlled trials comprising 1748 patients with head and neck cancer, established that PBM substantially reduced the incidence of severe oral mucositis, demonstrating relative risks (RR) typically ranging from 0.30 to 0.50 compared to placebo or sham controls. Furthermore, PBM demonstrated a highly significant reduction in patient-reported oral pain scores, lowering mean pain visual analog scale (VAS) scores by 2.0 to 3.0 points (19). These clinical effects directly correlate with reduced opioid consumption, improved quality of life, and fewer radiation therapy treatment interruptions. This extensive evidence base enabled the Multinational Association of Supportive Care in Cancer/International Society of Oral Oncology (MASCC/ISOO) and the World Association for Laser Therapy (WALT) to issue parameter-specific clinical practice guidelines recommending PBM for the prevention of oral mucositis in hematopoietic stem cell transplantation and head and neck cancer patients receiving radiotherapy or chemoradiotherapy (16, 17, 20).

### 3.2 Oropharyngeal mucositis: the anatomical frontier

The oropharynx highlights a primary limitation of conventional intraoral PBM: anatomical accessibility. While the anterior oral mucosa can be directly illuminated, the base of the tongue, tonsillar regions, soft palate, and pharyngeal walls are technically difficult to target with conventional point-probes. This challenge has driven interest in extended intraoral applicators, divergent wide-field sources, extraoral irradiation, combined intraoral–extraoral protocols, and anatomically shaped arrays. These approaches align with the dosimetric considerations described in supportive-care frameworks, emphasizing that anatomical coverage must become a formal dosimetric variable (20–22).

## 4 Xerostomia, hyposalivation, and salivary gland PBM

Radiotherapy-induced salivary dysfunction is a primary determinant of long-term oral morbidity in head and neck cancer survivors. PBM directed toward the major and minor salivary glands has produced encouraging results, although the clinical evidence remains more heterogeneous than for mucositis (19, 23–25). These applications are critical for device development because salivary glands represent significantly deeper targets than the superficial mucosa. Consequently, the dosimetric paradigm shifts from surface-delivered irradiance to attenuated target-tissue photon energy, making salivary PBM an important model for future tissue-specific depth dosimetry.

## 5 Beyond supportive oncology: PBM across oral health

To enhance readability and synthesize the highly variable data outside supportive oncology, the clinical characteristics, delivery geometries, and limitations of PBM across these secondary indications are summarized in Table 1.

**Table 1:** Potential indications of photobiomodulation in oral diseases.

Indication	Biological Rationale	Delivery Geometry	Stated Advantages/Key Outcomes	Major Technical & Clinical Limitations
Periodontal Disease	Adjunctive anti-inflammatory effect; stimulation of fibroblasts and tissue repair. (2, 5)	Transgingival (external) or intrasulcular (fiber-optic tip insertion into the pocket).	Improvements in clinical attachment levels (CAL) and probing pocket depth reductions when combined with mechanical debridement. (40, 41)	Extreme parameter heterogeneity; transgingival vs. intrasulcular routes alter tissue optical properties completely.
TMD & Orofacial Pain	Analgesia via nociceptor modulation; microvascular expansion; muscle trigger point relaxation.	Point contact over the temporomandibular joint (TMJ) capsule or masticatory muscle trigger points.	Clinically relevant reductions in pain scores; measurable improvements in maximal jaw opening. (47, 48)	High diagnostic heterogeneity (muscular vs. articular pathology); failure to account for target depth variations.
Oral Surgery Recovery	Modulation of acute postoperative inflammation; acceleration of fibroblastic proliferation. (2, 11)	Broad surface illumination over the extraction socket or surgical flap region.	Reductions in postoperative edema, trismus, and acute pain following third molar extractions.	Poorly standardized timing of application relative to the chronological phases of wound healing.
Implant Dentistry	Enhancement of early osseointegration; osteoblast proliferation; localized bone remodeling.	Trans-osteal illumination during implant placement or transmucosal application post-surgically.	Promising increases in implant stability quotients (ISQ) and accelerated early bone healing. (44–46, 49–51)	High inconsistency in findings due to massive variations in cortical bone thickness and resultant beam attenuation.
Orthodontics	Acceleration of bone remodeling via alveolar osteoclast/osteoblast modulation; pain relief.	Multi-point mucosal illumination along the roots of actively moved teeth.	Potential reduction in total orthodontic treatment time due to accelerated tooth movement. (52)	Wavelengths and exposure schedules vary drastically; high dependency on individual patient biological response.
Inflammatory Mucosal Disorders	Analgesia via nerve conduction modulation; epithelial cell proliferation.	Non-contact or contact surface scanning directly over painful erosive or aphthous lesions.	Pain relief in recurrent aphthous stomatitis, burning mouth syndrome, and oral lichen planus comparable to steroids. (38, 39, 42, 43, 53)	Lack of standardized endpoints; reliance on subjective pain reporting; low certainty of long-term efficacy evidence.

### 5.1 Critical analysis of negative, null, and inconclusive evidence

To ensure a rigorous and balanced review, it is vital to acknowledge that clinical evidence for PBM is not universally favorable. A critical review of the literature reveals several high-quality randomized controlled trials that reported null or inconclusive outcomes, particularly in lower-maturity indications. In periodontal therapy, multiple split-mouth trials evaluating adjunctive PBM failed to show statistically significant long-term differences in clinical attachment gain or microbiological profiles compared to scaling and root planing alone. Similarly, in implant dentistry, several studies found that while PBM might slightly accelerate early bone matrix deposition, it yielded no significant difference in final implant survival rates or secondary stability margins at 6 or 12 months post-loading. In orthodontics, multiple well-controlled trials concluded that PBM did not clinically accelerate canine retraction or alleviate initial alignment pain to a degree that justifies daily clinical intervention. These conflicting outcomes are rarely attributable to a failure of photobiological mechanisms *in vitro*; rather, they highlight the profound impact of negative publication bias, small sample sizes, and uncalibrated clinical delivery where the actual energy reaching the target tissue was insufficient to trigger a therapeutic response.

## 6 Why devices matter

The umbrella term “photobiomodulation device” encompasses technologies with fundamentally distinct optical characteristics. Two separate systems labeled as “810-nm devices” may differ markedly across multiple parameters:

- Laser versus LED emission sources
- Spectral bandwidth and beam divergence
- Spatial irradiance distribution and spot size
- Pulse structures (frequency, duty cycle)
- Thermal behaviors and field uniformity (3, 10).

Accordingly, nominal wavelength alone cannot define therapeutic equivalence. The historical laser versus LED debate is increasingly obsolete; the relevant question is whether a specific device configuration reliably delivers the required optical exposure to the intended biological target with accuracy, reproducibility, and safety (1, 3).

## 7 Device evolution in oral photomedicine

- Point-based laser systems: Establish the historical foundation of clinical PBM. They offer precise localization but suffer from operator dependence, long treatment times for large areas, and potential coverage gaps.
- Scanning and expanded-beam devices: Automate mucosal coverage to improve clinical efficiency, though source-to-tissue distance and scanning speed introduce new variables into dose management.
- Large-area LED arrays: Permit rapid, broad-field transcutaneous or mucosal illumination, depending heavily on emitter density, distance, and spatial uniformity.
- Anatomically adapted intraoral systems: Represent a major paradigm shift (e.g., mouthguard-style matrices). They provide reproducible source-to-tissue distances, simultaneous full-arch coverage, and minimized operator error.
- **Combined intraoral–extraoral platforms:** Essential for targeting complex regions like the oropharynx or salivary glands, combining transcutaneous and transmucosal photon delivery. This evidence base represents the exact same clinical data discussed in supportive oncology (20–22), but evaluated specifically through an engineering lens to overcome structural tissue barriers.
- Multi-wavelength devices: Blend red and NIR wavelengths to exploit complementary depth profiles, though each configuration requires independent physical and clinical validation.
- Home-based devices: Offer enhanced patient compliance for chronic conditions but mandate rigorous integrated dosimetry, output stability, and tracking to prevent misuse.
- Sensor-integrated smart devices: The next frontier, incorporating real-time distance, contact, temperature, and optical tissue feedback to transition PBM into an adaptive, closed-loop intervention.

## 8 Dosimetry: the central translational challenge

Reporting PBM “dose” solely in J/cm^2^ (radiant exposure) is insufficient (10). A reproducible clinical protocol requires the comprehensive reporting of at least 15 parameters, categorized across physical, temporal, and clinical dimensions (Table 2).

**Table 2:** Photobiomodulation parameters.

Category	Parameter	Standard Unit	Clinical & Reporting Significance
Optical Properties	Wavelength	nm	Dictates target tissue absorption and optical penetration depth. (3, 10)
	Spectral Bandwidth	nm	Defines the emission envelope (narrow for lasers, broad for LEDs).
	Beam Divergence	degrees	Dictates the rate of beam expansion and irradiance decay over distance.
	Beam/Spot Area	cm^2^	Crucial denominator required to accurately calculate irradiance.
Power Dynamics	Optical Power	mW (or W)*	Rate of energy delivery; depends on device class (mW for probes, W for broad arrays).
	Irradiance	mW/cm^2^	Instantaneous power density delivered to the illuminated surface.
	Emission Mode	Text (CW/Pulsed)	Defines whether light delivery is continuous or structurally interrupted.
Temporal Settings	Pulse Frequency	Hz	Dictates the modulation rate of pulsed delivery modes.
	Duty Cycle	%	The percentage of active “on” time during a single pulse cycle.
	Exposure Time	s	Duration of illumination per specific anatomical site.
Delivery Geometry	Total Energy	J	Total cumulative optical energy emitted (Power×Time).
	Radiant Exposure	J/cm^2^	Surface energy density delivered (Irradiance×Time).
	Source-to-Tissue Distance	mm or cm	Governs actual surface spot size and surface power density.
	Delivery Mode	Text (Contact/Non-contact)	Contact minimizes reflection and bypasses air interface dispersion.
Clinical Scheduling	Anatomical Sites	Count/cm^2^	Defines total surface area or volume targeted during the session.
	Session Frequency	Treatments/week	Governs biological recovery intervals between stimulations.
	Cumulative Exposure	Total sessions	Total longitudinal energy volume delivered over a clinical course.

*Optical power can be reported in milliwatts (mW) for focal surgical lasers/probes or watts (W) for high-output broad arrays.

## 9 The four levels of dosimetry

To resolve current translational ambiguities, we propose distinguishing four discrete levels of dosimetry (Table 3).

**Table 3:** The four levels of dosimetry for PBM across oral health.

Level	Formal Definition	Standard Assessment Method	Primary Source of Uncertainty
1. Device Output	Total optical emission directly at the emitter source.	Integrating sphere; power meter calibration.	Diode degradation; thermal drift; calibration stability. (3, 10)
2. Tissue-Surface Dose	Optical exposure actively reaching the mucosal/skin surface.	Calculation based on distance and beam profile.	Patient movement; handpiece angulation; operator variance.
3. Target-Tissue Dose	Attenuated photon energy reaching the deep biological target.	Derived via computational mathematical modeling.	Spatial scattering; blood perfusion; localized bone density. (9)
4. Biological Dose	Downstream cellular and metabolic stimulus generated by light.	Quantification of localized biomarkers or clinical response.	Biphasic limits; patient phenotype; tissue health baseline. (26)

**Table 4:** Clinical maturity and evidence positions of PBM across oral health indications.

Indication	Clinical Maturity Rating	Primary Intended Outcome	Principal Unresolved Translational Issue
Oral Mucositis	High	Incidence and severity reduction of Grade 3–4 lesions. (14–17, 19)	Standardization of protocol transfer across distinct device classes.
Oropharyngeal Mucositis	Moderate/Emerging	Complete coverage of deep posterior pharyngeal walls. (20–22)	Physical anatomical access and verified target-tissue dose delivery.
TMD/Orofacial Pain	Moderate/Heterogeneous	Articular and muscular pain reduction; functional recovery. (47, 48)	Lack of deep target differentiation and strict diagnostic phenotyping.
Xerostomia/Hyposalivation	Emerging/Moderate	Salivary gland preservation and active flow recovery. (19, 23–25)	Deep glandular dosimetry modeling through overlaying tissues.
Periodontitis	Emerging	Adjunctive pocket inflammation reduction and attachment gain. (40, 41)	High protocol heterogeneity; transgingival vs. intrasulcular variance.
Oral Surgery Recovery	Emerging	Reductions in postoperative pain, trismus, and edema. (2, 11)	Unclear chronological timing relative to specific healing phases.
Dental Implants	Emerging/Inconsistent	Acceleration of early bone matrix deposition and stability. (44–46, 49–51)	High beam attenuation through varying cortical bone thicknesses.
Orthodontics	Emerging	Accelerated tooth movement; relief of initial archwire pain. (52)	Low reproducibility; lack of synchronized exposure schedules.
Inflammatory Mucosal Disorders	Exploratory/Emerging	Direct analgesia and accelerated ulcer/lesion healing. (38, 39, 42, 43, 53)	Inconsistent clinical endpoints; reliance on subjective reporting.

In oral anatomy, photon propagation is highly influenced by localized variables: melanin, hemoglobin, water content, mucosal thickness, keratinization, saliva, blood, and bone density (9). Consequently, identical device output settings do not guarantee identical biological doses across different patients or anatomical regions.

## 10 Geometry is part of the dose

Oral anatomy creates a unique dosimetric problem. The oral cavity contains curved, irregular, mobile, and optically heterogeneous surfaces. A probe positioned perpendicular to buccal mucosa does not create the same exposure as the identical probe directed obliquely toward the posterior tongue. For divergent light sources, distance changes irradiance; for non-contact treatment, the angle of incidence changes effective surface exposure; and for large arrays, overlapping emission fields determine spatial uniformity. Therefore, treatment geometry should be regarded as a primary dosimetric parameter rather than an operational detail (3, 9).

## 11 The biphasic response: avoid over-dosing

PBM operates on a biphasic tissue response curve (Arndt-Schulz law), where insufficient optical energy fails to trigger a response, while excessive exposure can suppress or reverse the desired therapeutic effects (26). The goal of precision photomedicine is not to deliver the maximum tolerated dose, but to pinpoint the optimal biological exposure window. Future dose-finding studies must investigate response surfaces involving irradiance, radiant exposure, treatment intervals, and tissue characteristics rather than single-variable escalation.

## 12 Calibration and quality assurance

One of the under-recognized challenges in PBM is the assumption that displayed device settings correspond exactly to actual optical output. Regular quality assurance protocols should verify wavelength, optical power, irradiance, temporal stability, uniformity across multi-emitters, pulse characteristics, and thermal behavior. This becomes particularly important in multi-emitter devices, where degradation or variability between individual sources may create heterogeneous treatment fields. Rigorous calibration and complete reporting are prerequisites for meaningful comparison between clinical protocols (3, 10).

## 13 Computational photon-transport modeling

The next major advance in biophotonics will rely heavily on numerical computational modeling to map photon propagation through complex, multilayered structures. While tissue optics principles are well-established (9), translating them into patient-specific dosimetry represents an active research frontier. Historically, Monte Carlo simulations have been utilized extensively in biomedical optics to map light distribution in highly scattering media, such as dermatology and neuroimaging (27, 28). In oral medicine, these numerical models must be adapted to calculate how light attenuates across stratified squamous epithelium, vascularized lamina propria, submucosal fat, muscle, and dense cortical bone. By inputting wavelength-dependent absorption (*μ*a) and scattering (μs) coefficients—heavily influenced by local concentrations of hemoglobin, water, and melanin—computational frameworks can predict volumetric energy deposition (9, 29). Rather than prescribing rigid surface energy densities (J/cm^2^) at the probe tip, clinicians could utilize these predictive algorithms to calculate the exact optical power necessary to achieve a therapeutic threshold at deep targets, such as the minor salivary glands or underlying alveolar ridges.

## 14 Biomarkers and optical monitoring

A current limitation of PBM clinical execution is the “blind” delivery of light, wherein clinicians cannot verify whether a threshold biological response has been achieved. Precedents in other phototherapies, such as real-time optical coherence tomography (OCT) or diffuse reflectance spectroscopy (DRS) in photodynamic oncology, suggest that tissue feedback can dramatically reduce clinical variance (30, 31). The highly accessible oral cavity is uniquely positioned to adopt integrated optical monitoring systems. Future smart handpieces could pair therapeutic laser diodes with real-time diagnostic sensors to track immediate tissue changes. This includes monitoring rapid physical shifts—such as localized microvascular blood flow velocity via laser Doppler flowmetry, or changes in tissue oxygenation (StO₂) (32). Concurrently, biochemical tracking via rapid chairside salivary diagnostics could measure immediate post-illumination changes in inflammatory cytokines (e.g., IL-1β, TNF-α) or oxidative stress biomarkers. Merging therapeutic delivery with real-time physiological feedback will allow clinicians to objectively determine when a sufficient biological dose has been achieved.

## 15 Artificial intelligence and precision oral photomedicine

Artificial intelligence (AI) and machine learning architectures could serve as the foundational integration layer for precision oral photomedicine. Analogous to AI-driven adaptive radiation therapy planning, which continually recalculates ionizing radiation fields based on daily anatomical shifts, machine learning models could optimize non-ionizing PBM dosimetry (33, 34). An AI algorithm could process an integrated patient data matrix, combining baseline clinical phenotypes, anatomical tissue mapping (derived from intraoral optical scans), localized biomarker profiles, and device performance histories. By cross-referencing this information with computational photon models, the AI could execute real-time closed-loop adjustments. For example, if an intraoral sensor detects an increase in mucosal blood pooling or patient movement during irradiation, the algorithm could instantly modulate device output power, pulse frequency, or duty cycle to maintain a steady target-tissue dose. This transitions PBM from a fixed, device-centered protocol into a highly dynamic, individualized photonic intervention.

## 16 Comprehensive safety and risk assessment

### 16.1 Oncology vigilance and safety

PBM in oncology requires particular attention because some intracellular signaling pathways modulated by light—such as the upregulation of ATP, activation of transcription factors, and modulation of reactive oxygen species—are also relevant to malignant cell biology (21). Preclinical *in vitro* observations are highly heterogeneous and strongly dependent on cell lines, precise wavelengths, and irradiances; these laboratory findings cannot be directly extrapolated to clinical recurrence or survival outcomes. Crucially, long-term clinical data from trials utilizing supportive-care PBM protocols for oral mucositis have demonstrated no adverse oncological signals, and international expert organizations strongly support its use in defined oncology settings (16, 17, 20). Nevertheless, prospective surveillance remains essential. Registries must systematically document cancer types, exact tumor boundaries, irradiated fields, and long-term recurrence or progression outcomes. The appropriate clinical stance remains evidence-based oncological vigilance rather than either theoretical prohibition or indiscriminate reassurance.

### 16.2 General safety, contraindications, and home-use risks

Beyond oncology, a rigorous clinical framework must incorporate general laser safety, thermal monitoring, and a clear understanding of absolute and relative contraindications. Ocular Safety: The primary hazard in PBM clinical delivery is accidental ocular exposure. Direct or specularly reflected beams from visible red or NIR lasers can cause irreversible retinal photocoagulation or thermal damage (35). Strict adherence to laser safety standards—including the mandatory use of wavelength-specific protective eyewear for the clinician, patient, and assistants—is absolute. Thermal Hazards: While PBM is defined as a nonthermal therapy, multi-emitter LED arrays or high-power density laser probes can generate localized surface heating, particularly when operating in continuous wave mode over highly pigmented tissues or metallic restorations. Irradiance must be kept below thermal thresholds to prevent accidental tissue coagulation or accelerated inflammatory responses. Contraindications & Photosensitization: PBM should be avoided directly over active primary malignant lesions (unless part of an approved supportive protocol), active systemic lupus erythematosus (SLE) lesions, or the gravid uterus. Furthermore, clinicians must thoroughly screen patients for concurrent use of systemic photosensitizing medications (e.g., amiodarone, tetracyclines, thiazides), which can radically lower the skin or mucosa's threshold for phototoxic injury (36). Home-Use Device Risks: The commercial expansion of patient-administered home PBM devices introduces distinct safety concerns. Unsupervised patients face risks of accidental ocular exposure due to lack of standard safety goggles, thermal injury from prolonged application over a single spot (“more is better” misunderstanding), and therapeutic failure due to uncalibrated battery drift or incorrect placement. Consequently, home photomedicine demands highly robust device engineering, including integrated contact sensors, auto-shutoff timers, and mandatory clinician supervision.

## 17 Research priorities

Future biophotonics research must shift from generic efficacy inquiries (“does light work?”) toward precise multi-variable questions. Key clinical research priorities include:

- Standardized Reporting: Implementing mandatory reporting checklists covering all 15 parameters outlined in Table 2 (3, 10).
- Independent Calibration: Validating actual device optical power and spatial uniformity separate from manufacturer specifications.
- Dose-Ranging Trials: Performing rigorous mapping of the biphasic response curve across distinct biological phenotypes (26, 37).
- Computational Integration: Developing accessible clinical software for real-time Monte Carlo tissue optical mapping (9).
- Prospective Registries: Constructing multi-center databases to monitor long-term oncological safety and secondary clinical outcomes.

## 18 From standardized PBM to precision oral photomedicine

Standardization and personalization are not opposing objectives; standardization is the absolute prerequisite for personalization. Without standardized terminology, physical parameters, calibration, and outcome measures, reliable individualized treatment cannot be achieved. The evolution of the field can be structured across four defined paradigms:

1. PBM 1.0 (Device-Centered): Characterized by fixed manufacturer settings and manual point-based handpiece application.
2. PBM 2.0 (Protocol-Centered): Characterized by evidence-based, indication-specific surface parameters (J/cm^2^).
3. PBM 3.0 (Anatomy-Centered): Characterized by broad anatomical coverage arrays and integrated tissue optical attenuation modeling.
4. Precision Oral Photomedicine (Patient-Centered): The definitive synthesis, integrating real-time anatomy, dynamic tissue optical properties, continuous biomarker monitoring, and AI-driven adaptive closed-loop dosimetry.

## 19 Conclusion

Biophotonics is successfully evolving from a disparate collection of light-based technologies into a coherent therapeutic and diagnostic framework for oral healthcare. The clinical success of PBM in oral mucositis provides definitive proof that rigorous photonic research can generate guideline-supported interventions. The major challenge of the coming decade will not be the discovery of an isolated “magic” wavelength, but the paradigm transition from emitted device output to dynamic target-tissue dose. By bridging light-based innovation with advanced computational physics, clinical biomarkers, and adaptive engineering, oral healthcare can become a leading medical domain in the realization of genuinely personalized precision photomedicine (Table 4).

## 20 Figures and structural content verification

Figure 1 Verification Statement: The technical content of Figure 1—depicting the precision oral photomedicine translational pathway (incorporating the wavelength axes, physical dosimetric units, tissue layering, and the sequential closed-loop AI dosimetry flow)—has been independently evaluated, revised, and verified by the authors for absolute scientific and physical accuracy.

**Figure 1:**
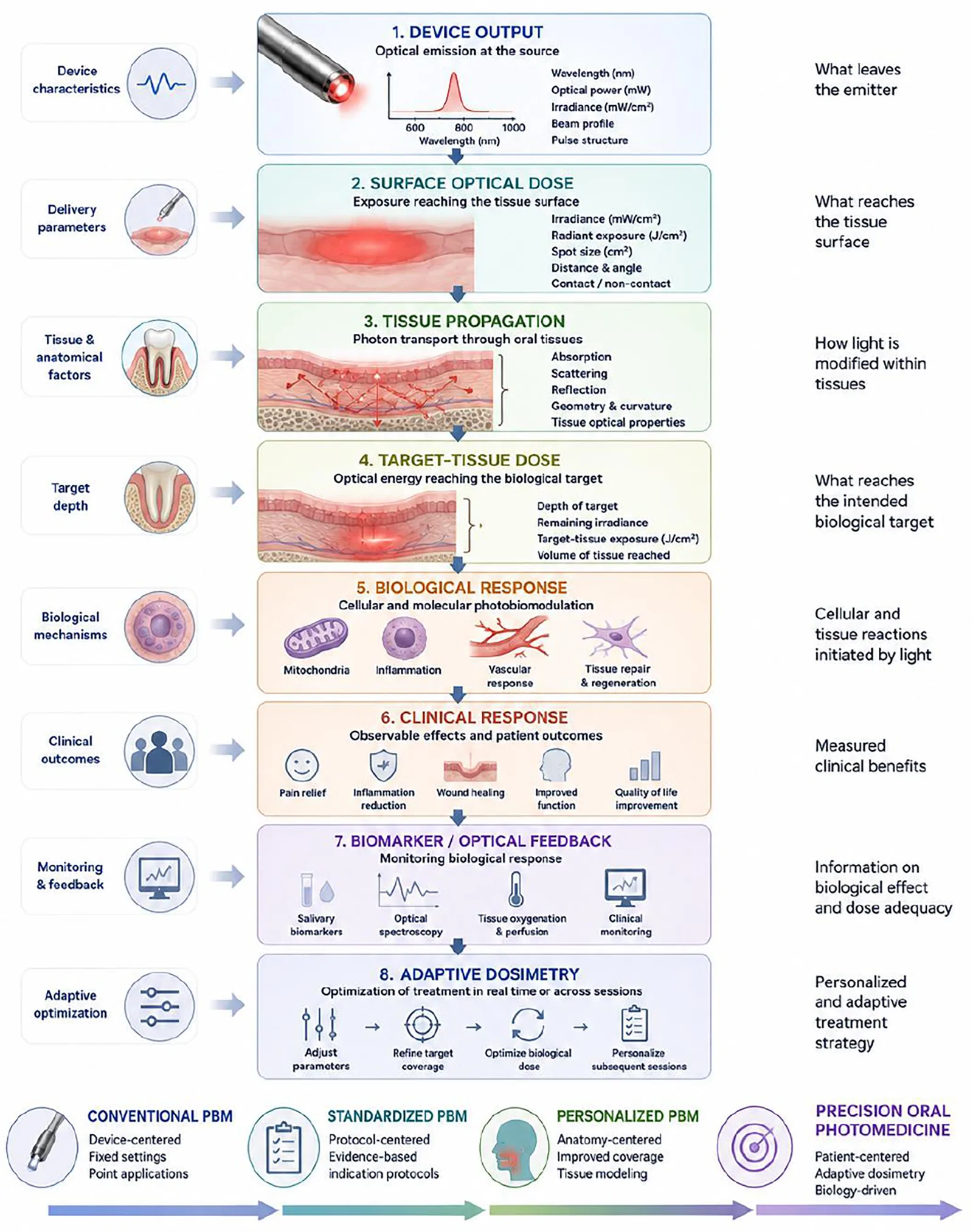
Photomedicine in Oral Health.
